# Resolving the differential distribution of structural proteins in baculovirus using single-molecule localization microscopy

**DOI:** 10.1099/jgv.0.002054

**Published:** 2024-12-02

**Authors:** Daniel Martínez-Flores, Aaron Pavel Rodríguez-Hernández, Alicia Sampieri, Adolfo Cruz-Reséndiz, Ileana Tobías-Juárez, Reyna Lara-Martínez, Luis F Jiménez-García, Luis Vaca

**Affiliations:** 1 Departamento de Biología Celular y del Desarrollo, Instituto de Fisiología Celular, Universidad Nacional Autónoma de México, Ciudad Universitaria, México City 04510, Mexico; 2 Departamento de Microbiología y Parasitología, Facultad de Medicina, Universidad Nacional Autónoma de México, Ciudad Universitaria, México City 04510, Mexico; 3 Departamento de Biología Celular, Facultad de Ciencias, Universidad Nacional Autónoma de México, México City 04510, Mexico

**Keywords:** baculovirus, E25, GP64, P24, single-molecule localization microscopy, structural proteins

## Abstract

Baculovirus is one of the most complex viruses found in nature. Proteomic analysis of budded viruses (BVs) indicates that they are formed by at least 50 different structural proteins. The function of most of these structural proteins and their specific localization in individual virions remain unknown. In the present study, we have conducted single-molecule localization microscopy analysis of the spatial distribution of the nucleocapsid protein P24 and the envelope proteins GP64 and E25. Our results show that P24 and GP64 are polarized to one end of the baculovirus, while E25 distributes more homogenously along the viral envelope. This is the first study using optical microscopy to demonstrate the polarized distribution of structural proteins in individual baculoviruses.

## Introduction

Although the nanometric size of viruses may suggest low complexity, viruses are structurally and functionally sophisticated and highly diverse as evidenced by electron microscopy studies [1]. In 1939, the first electron microscopy study of the tobacco mosaic virus was reported [2]. This was the first virus visualized by transmission electron microscopy (TEM). This microscopy method has become the gold standard to visualize viruses with high resolution [3,4], but recent advances in optical microscopy are providing a different view of viruses with higher resolution than before [5]. Conventional optical systems have a resolution limit established by a physical barrier, the light diffraction limit [6]. This fundamental limit was early recognized by the physicist Ernst Karl Abbe in 1873 [7]. However, recent advances in what is now called super-resolution microscopy are pushing the limits to higher standards [8,10].

Optical microscopy provides many advantages over TEM for the study of virus structures [8]. TEM allows the visualization of structures with very high resolution, but identifying the proteins responsible for such structures has been challenging to this date. Using fluorescence microscopy in combination with selective labelling techniques using antibodies or generating viral proteins fused to fluorescent proteins provides effective tools for identifying proteins that form specific viral structures [11,17].

An optical method of super-resolution microscopy that provides very high resolution is known as single-molecule localization microscopy (SMLM) [18]. This method operates by differentiating individual fluorophores in diffraction-limited samples. High-intensity excitation light can bring fluorophores into fluorescence intermittency or blinking, resulting in the random switching between ON (bright) and OFF (dark) states of the fluorophores under continuous excitation [18]. Gathering thousands of images from a specimen captures individual blinking fluorophores, which are used to reconstruct a high-resolution image of the entire specimen [8,9, 18]. SMLM is a general term, which includes methods such as stochastic optical reconstruction microscopy (STORM) and photo-activated localization microscopy (PALM). Both methods are based on the same basic principles described above, but typically studies refer to STORM when the fluorophores are conjugated to antibodies that recognize the protein of interest, while the term PALM refers to the use of fluorescent proteins [9].

Super-resolution microscopy methods (SMLM and Stimulated Emission Depletion Microscopy) have provided very relevant information about the different protein layers within individual herpes simplex virus 1 particles, differentiating envelope-associated glycoproteins from tegument proteins [13,14]. A recent study using SMLM to analyse individual viruses identified a greater axial symmetry in terms of protein distribution in Severe acute respiratory syndrome coronavirus 2 compared to influenza viruses [17].

Insect DNA viruses from the *Baculoviridae* family infect different arthropods but mainly lepidopteran larvae [19]. Baculoviruses are typically 40–60 nm wide and 230–385 nm in length, with a genome formed by a double-stranded circular DNA [20]. TEM studies combined with immunogold have shown that the GP64 glycoprotein is polarized towards one end of the baculovirus [19,21,23]. This protein is essential for insect cell infection and virus entry into the host cell [19]. However, proteomic analysis of budded viruses (BV) has revealed over 50 structural proteins of which nothing is known about their spatial distribution [24]. Most of these structural proteins have unknown function.

In the present study, we utilized SMLM to study the spatial distribution of P24, a nucleocapsid-associated protein [25], and E25, an envelope protein [26,27] from the baculovirus *Autographa californica nucleopolyhedrovirus* (AcMNPV). We demonstrate for the first time using optical microscopy that GP64 and P24 structural proteins are in one pole of the baculovirus (in closed proximity to each other), while the envelope protein E25 distributes more homogeneously along the baculovirus envelope. The polarized localization of GP64 was previously evidenced using TEM-immunogold [21]. Our study demonstrates that SMLM is a powerful tool capable of identifying spatial gradients of structural proteins in single baculoviruses. The polarized spatial distribution of some structural proteins may have functional implications in the baculovirus biology. Future studies forcing delocalization of GP64, P24 or any other structural protein of interest may provide insights into their location–function relationships. Mapping the spatial distribution of all baculovirus structural proteins may provide a detailed roadmap of how one of the most sophisticated viruses in nature is assembled.

## Methods

### Construction of recombinant baculoviruses and cell culture

The genes encoding for the E25 (GeneID: 1403927) and P24 (GeneID: 1403962) proteins of *AcMNPV* were cloned into the pFastBac™ transfer vector (Thermo Fisher Scientific, USA) under the control of the polyhedrin promoter (p*PH*) and in the reading frame with the monomeric variant of the EGFP gene (Takara Bio USA, San Jose, CA). Cloning was performed using restriction enzymes and confirmed by sequencing.

The pFastBac™ transfer plasmid containing the constructs was used to perform transposition in DH10Bac™ *Escherichia coli* cells, which contain the bacmid and the helper plasmid, as part of the Bac-to-Bac® system (Thermo Fisher Scientific). *Spodoptera frugiperda* cells (Sf9 ATCC®, USA, cat. no. CRL-1711, RRID: CVCL_0549) were transfected with the bacmid for the generation of recombinant baculoviruses using the transfecting agent FlyFectin according to the manufacturer’s specifications (OZ Biosciences, San Diego, CA, USA, cat. No. FF50500).

The supernatant from the transfected cells was collected as the initial P1 virus, and Sf9 cells in the logarithmic growth phase (2×10⁶ cells ml^−1^) were infected with the P1 viral passage and incubated at 27 °C with constant shaking. After 72 h of infection, the supernatant was collected as the P2 viral passage. The process was repeated until reaching passage P3, with which large-scale expression was conducted to recover the virus from the supernatant.

Sf9 cells were maintained in Grace medium (Thermo Fisher, USA, cat. no. 11300-027) supplemented with 10% inactivated FBS (Biowest, France, cat. no. S1650-500), lactalbumin (Sigma-Aldrich, USA, cat. no. 19010), yeastolate (Thermo Fisher, cat. no. 292805), antibiotic–antimycotic (Thermo Fisher, cat. no. 15240-062) and 0.1% pluronic acid F-68 (Sigma-Aldrich, USA, cat. no. P1300) at 27 °C under stirring.

### Purification of baculoviruses

Recombinant baculoviruses were produced using Sf9 insect cells. Incubation was carried out at 27 °C with 100 r.p.m. shaking until a volume of 50 ml was reached at a density of 2×10⁶ cells ml^−1^. Subsequently, each flask was infected with recombinant baculoviruses BacE25-EGFP and BacP24-EGFP, as well as BacWT. After 5 days post-infection, cells were recovered and centrifuged at 2435 ***g*** to precipitate cells in SLA-1500 rotor flasks for 10 min at 4 °C. The supernatant was recovered and centrifuged at 103 864 ***g*** in a SW-28 rotor for 1.5 h at 4 °C to concentrate the budded baculoviruses. The pellet was then left in 2 ml of sterile PBS overnight at 4 °C. The next day, resuspended budded baculoviruses were recovered and loaded onto a 50 and 10% sucrose gradient in SW-28 rotor tubes; baculoviruses were isolated from the gradient interface. To remove excess sucrose, viruses were centrifuged again under the same parameters in sterile PBS. The supernatant was discarded, and they were kept in PBS overnight at 4 °C. Finally, budded baculoviruses were resuspended, recovered and stored at −70 °C until use.

### Western blotting

**Fig. 1.**
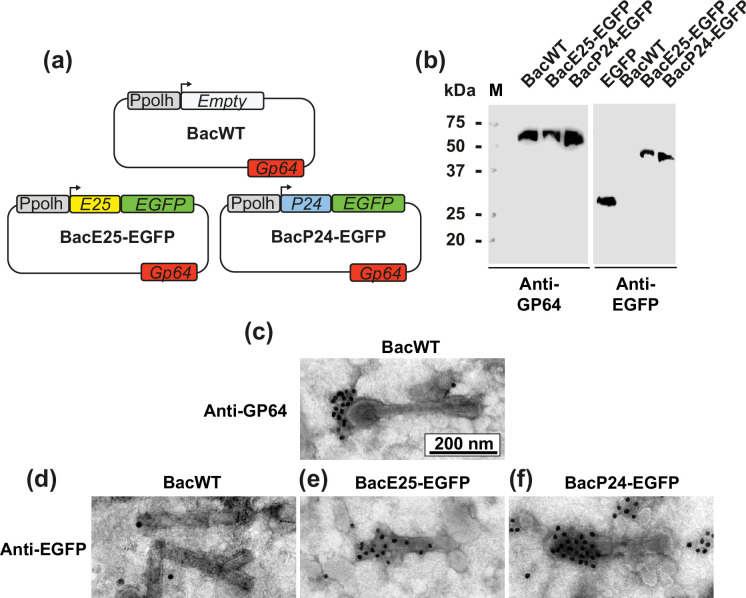
Recombinant budded baculoviruses express and display P24-EGFP and E25-EGFP. (**a**) Schematic representation of the genetic construction of wild-type (BacWT) and recombinant baculoviruses E25-Egfp (53.1 kDa) (BacE25-EGFP) and P24-Egfp (49.14 kDa) (BacP24-EGFP). Polh (grey box) is the promoter used for the expression of E25-EGFP and P24-EGFP. All viruses express the endogenous GP64 glycoprotein (red box). (**b**) Western blotting for the immunolabelling of GP64 and EGFP in purified BV. BacWT was used as negative controls for anti-EGFP. (**c**) BacWT baculoviruses were immunolabelled to detect the endogenous GP64 glycoprotein. (**d**) BacWT used as a negative control to show no recognition of EGFP in wild-type baculovirus. This was the control for primary and secondary antibodies. (**e**) Identification in a single baculovirus of E25-EGFP in the recombinant BacE25-EGFP baculovirus. (**f**) Identification of P24-EGFP in a single recombinant BacP24-EGFP baculovirus. Secondary antibodies are conjugated to 15 nm gold nanoparticles (Methods).

Purified BacWT, BacP24-EGFP and BacE25-EGFP baculoviruses and control protein EGFP (previously produced in our laboratory [28]) were mixed with Laemmli buffer (50 mM Tris-HCL, 3% SDS, 1% β-mercaptoethanol, 20% glycerol and 0.7% bromophenol blue and pH 6.8) and loaded onto a 10% SDS-PAGE gel (Fig. 1b). Subsequently, the proteins were transferred to nitrocellulose membranes (Merck Millipore, USA, cat. no. HATF00010).

The membrane was blocked with TBS+5% fat-free milk for 1 h at room temperature. It was then cut and incubated with two different primary antibodies: anti-GP64 (1 : 2000) (Thermo Fisher, cat. no. 14-6995-82) and anti-GFP (1 : 500) previously produced in our laboratory [29]. Primary antibodies were incubated at 4 °C under shaking overnight. After washing the membranes, they were incubated with the secondary antibody HRP-coupled anti-mouse IgG (1 : 5000; Sigma-Aldrich, cat. no. A9044). The membranes were washed again, and SuperSignal® West Pico Plus substrate (Thermo Fisher, cat. no. 34577) was added. Image acquisition was performed with a C-DiGit Blot scanner (LI-COR, USA).

### TEM-immunogold

For immunoelectron microscopy, BacWT, BacP24-EGFP and BacE25-EGFP were incubated with anti-GP64 (1 : 10) or anti-EGFP antibody (1 : 10, Takara, JP, cat. no. 632592) overnight at 4 °C, subsequently bound to Formvar-coated gold grids, immunolabelled with gold-conjugated antibody (1 : 10, 15 nm in diameter, Aurion, NL cat. no. AU25816) overnight and washed with PBS. The grids were contrasted with 4% uranyl acetate for 5 min and then with 0.4% lead citrate for 3 min at room temperature. The grids were washed with distilled water and air-dried before visualization in a JEM-1010 TEM (JEOL, Peabody, MA, USA), operating at 80 kV. Image acquisition was performed using the Gatan Microscopy Suite software (V. 2.32.888.0).

### Production of anti-P24 antibodies

The recombinant P24-His(6 x) protein was expressed and purified from *E. coli* BL21(DE3). P24-His(6 x) was purified using affinity chromatography and dialyzed against PBS. The purity and identity of the protein were confirmed by SDS-PAGE.

Six- to eight-week-old BALB/c mice were immunized with the purified P24-His(6×) protein in a 1 : 1 mixture with aluminium hydroxide. Each mouse received an intramuscular injection (100 µl) with a concentration of 15 µg of protein per mouse. The mice were immunized twice: the first on day 0 and the second application 2 weeks later. Serum obtained by submandibular puncture from immunized mice was collected before immunization (baseline control) and at 14-day intervals.

The production of specific antibodies against the P24 protein was evaluated using ELISA. Ninety-six well plates (Corning, USA, cat. no. 3590) were coated with P24 (5 µg ml^−1^ in PBS) and incubated overnight at 4 °C. After blocking with milk in PBS-T (PBS with 0.05% Tween-20) for 1 h at 37 °C, the plates were washed with PBS-T, and mouse serum was added. Specific antibodies were detected using a mouse anti-IgG secondary antibody conjugated with HRP and revealed with the 3, 3', 5, 5' - Tetramethylbenzidine substrate solution. The reaction was stopped with 0.16 M H₂SO₄, and the absorbance was measured at 450 nm using a multiskan FC 3.1 microplate reader (Thermo Fisher).

### Labelling and mounting of viruses

The viral envelope was fluorescently labelled using the CellBrite™ Steady 650 (Biotium, USA, no. cat. #30108 T) membrane labelling probe during the purification process. Purified viral particles were resuspended in a labelling solution containing CellBrite™ according to the manufacturer’s instructions. The mixture was incubated at room temperature for 30 min with gentle agitation to ensure uniform incorporation of the probe into the viral envelope. After labelling, the excess dye was removed by performing a series of purification steps, including sucrose gradient ultracentrifugation, to obtain the labelled viral particles. The integrity of the viral envelope was assessed by TEM.

Recombinant baculovirus particles expressing GP64 and P24/P24-EGFP were subjected to immunofluorescence labelling to visualize the distribution of these proteins. Viral particles were mounted on coverslips pre-coated with poly-l-lysine (0.01%), and the samples were blocked with 3% BSA in PBS to prevent non-specific binding.

Primary antibodies specific to GP64 (anti-GP64, 1 : 100) and P24 (anti-P24, 1 : 100) were incubated for 3 h at room temperature. The samples were then washed with PBS and incubated with fluorophore-conjugated secondary antibodies (CF568, 1 : 100, Biotium, no. cat. #20100) for 1 h at room temperature in the dark. After additional washes to remove excess secondary antibodies, the labelled viral particles were mounted on slides and analysed by SMLM.

For SMLM imaging, a blinking buffer was prepared containing 100 mM mercaptoethylamine (Sigma-Aldrich, cat. no. #30070-50G), 8.9% glucose (Sigma-Aldrich, cat. no. G5767-500G), 0.56 mg ml^−1^ glucose oxidase (Sigma-Aldrich, cat. no. #G2133-250KU) and 170 µg ml^−1^ catalase (Sigma-Aldrich, cat. no. #C40-100MG). The sample was mounted with the blinking buffer between the slide and coverslip containing the sample and sealed with nail polish to prevent oxidation during acquisition.

### SMLM imaging

Images were acquired using an inverted ONI Nanoimager microscope (Oxford Nanoimaging Ltd., Oxford, UK) equipped with a 100× oil immersion objective lens, NA 1.4. The system is equipped with the ORCA-Flash 4.0 (V3 C133440-20CU) camera with a pixel size of 6.5×6.5 μm^2^. Fluorophores were excited using lasers with wavelengths of 405, 473, 561 and 640 nm. All four lasers have a power of 1 W measured at the source. The 405 nm laser was used to promote the initial fluorophore blinking for all acquisitions. Total internal reflection fluorescence illumination was employed to capture the fluorescence of viral particles closest to the coverslip. ONI Nanoimager microscope provides a resolution limit of 20 nm according to the manufacturer (Oxford Nanoimaging Ltd.). We conducted a parameter-free image decorrelation analysis, which provided a resolution limit of 6.6 nm under our experimental conditions [30].

Chromatic aberration correction was performed using 100 nm TetraSpeck™ microspheres (Thermo Fisher Scientific, cat. no. T7279), and the temperature control system was enabled to minimize drift during acquisition.

Image series were acquired for each field of view with an exposure time of 30 ms per frame for a total of 10 000 frames per channel. The precise localization of each fluorophore was identified in real time during image acquisition using the ONI, NimOS software (ONI v.1.19.5.20230223170946-e050349, Oxford Nanoimaging Ltd.). The acquired images were individually exported per channel with a pixel size of 0.5 nm in a compatible TIFF (Tag Image File Format) and imported into ImageJ (FIJI) software (v. 1.54 f, NIH, Bethesda, MD, USA) for further analysis.

### Fluorescence distribution analysis

The SMLM images, after being exported and imported into ImageJ, were analysed using the rectangular selection tool in the region of interest (ROI) of the image, where the viral particles had previously been horizontally aligned with the ImageJ software.

With the ROI selected, we utilized the ‘Plot Profile’ function from Fiji to calculate the average pixel intensity along the X-axis for each ROI. This process was repeated for the second fluorophore, ensuring that the acquisition and processing conditions were equivalent for both samples. Identical rectangles were drawn to ensure that the same areas were measured in the images corresponding to both fluorophores. Given the differences in lengths among the baculoviruses, the data were normalized as a percentage of length, and the fluorescence intensities were adjusted and presented as arbitrary fluorescence units.

### Molecule counting

For the analysis of the number of displayed molecules of E25-GFP and P24-GFP in individual baculoviruses, a script was developed in R studio (supplementary information, available in the online version of this article). Raw data were extracted from the images obtained for each SMLM acquisition with the NimOS software (Oxford Nanoimaging Ltd.). The raw data contained coordinates (X, Y pixel localization) for each particle and each fluorescence channel. The R script reads the millions of data points from each SMLM image, filters the data by channel and X and Y coordinates and performs the analysis of individual viruses, discarding molecules with the same coordinates to eliminate duplicated particles in the counting. The procedure was conducted for 15–25 individual baculoviruses for BacE25-EGFP, BacP24-EGFP and BacWT.

### Statistical analysis

The data obtained on the molecular counts for GP64-WT, E25-EGFP and P24-EGFP were analysed using a two-way ANOVA to compare the mean of each group and determine if there were significant differences. A post-hoc Tukey test was performed for multiple comparisons. Results were considered significant when the *P*-value was less than 0.05. To assess whether there was a significant difference between the molecular count means of P24 and P24-EGFP, a Student’s t-test was employed. Results were considered significant when the *P*-value was less than 0.0001.

## Results

### Generation of recombinant baculovirus for SMLM studies

For the present study, we generated two recombinant baculoviruses, one carrying a fusion of the nucleocapsid-associated protein, P24 to EGFP (BacP24-EGFP), and the other carrying a fusion of the envelope protein E25 to EGFP (BacE25-EGFP). The expression of both transgenes was driven by the strong polyhedrin promoter. As a control, we use a wild-type baculovirus (BacWT, Fig. 1a). The BacWT contained the same polyhedrin promoter but without the transgene (Fig. 1a, empty vector). Baculoviruses were generated using the baculovirus-insect cell expression system (Methods), amplified and purified before use.

Budded baculoviruses (BV) were purified and analysed by Western blot (Fig. 1b). First, we identified the GP64 glycoprotein since only BV carry this structural protein. As illustrated in the figure, all recombinant baculoviruses show high levels of GP64, indicating that they were BV (Fig. 1b). Using a commercially available monoclonal antibody against EGFP, we identified the fusion protein E25-EGFP in BacE25-EGFP and P24-EGFP in BacP24-EGFP. As a control, we use purified EGFP.

Next, we conducted TEM-immunogold analysis of the baculoviruses to confirm the presence of GP64 in BacWT (Fig. 1c) using the commercially available anti-GP64 antibody. To detect the recombinant proteins, we first used BacWT as a negative control with an anti-EGFP antibody (Fig. 1d). Next, we use the anti-EGFP antibody to identify E25-EGFP (Fig. 1e) and P24-EGFP (Fig. 1f) on the surface of the baculovirus.

### Distribution of E25 envelope protein on individual baculovirus

Once we were confident that we had purified BV and that each recombinant baculovirus carried their respective fusion protein (P24-EGFP and E25-EGFP), we proceeded to analyse individual virus particles with SMLM. For this purpose, we detected the fluorescence of EGFP, and for GP64, we use a monoclonal antibody, and a secondary antibody labelled with a CF568 fluorescent probe (Methods). This combination of fluorophores allows the simultaneous identification of GP64 and the recombinant protein (P24-EGFP or E25-EGFP) in the same viral particle.

**Fig. 2.**
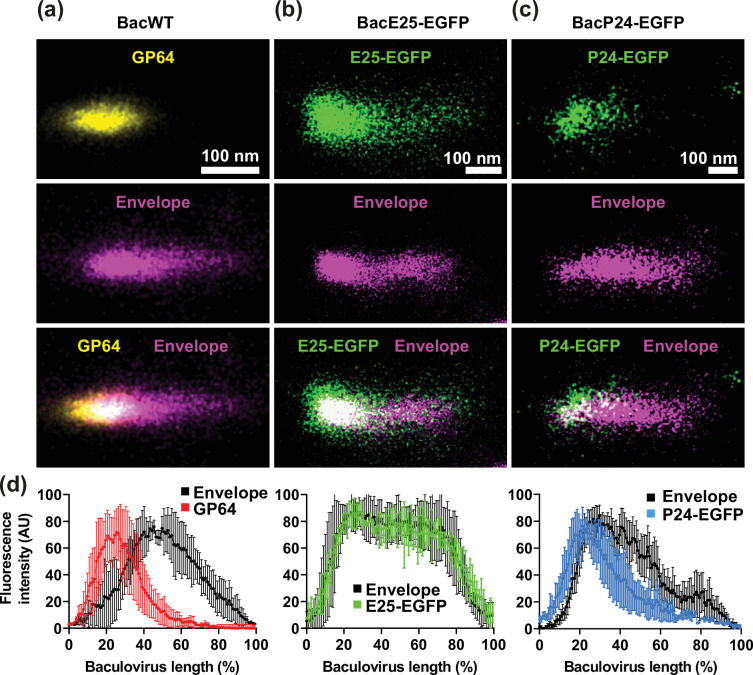
Spatial distribution of GP64, P24-EGFP and E25-EGFP proteins in budded baculoviruses in relation to the viral envelope. Purified BacWT (**a**), BacP24-EGFP (**b**) and BacE25-EGFP (**c**) baculoviruses were incubated with the fluorescent membrane dye CB650 for viral envelope labelling (purple). P24-EGFP and E25-EGFP are shown in green, while GP64 (yellow) was immunolabelled for fluorescence detection using the fluorescent dye CF568 (Methods). EGFP, CF568 and CB650 were excited with wavelengths of 473, 561 and 640 nm, respectively (Methods). UV light was utilized to induce fluorophore blinking (Methods). (**d**) Mean±sd obtained from at least 15 individual baculoviruses. The plot shows average fluorescence distribution along the baculovirus length (mean±sd) for GP64, E25-EGFP and P24-EGFP. To visualize the limits of each individual baculovirus, the membrane fluorescent marker CB650 was utilized to label the viral envelope (Methods). Baculovirus lengths were normalized to percentage for easier comparison, since baculoviruses have a range of lengths.

Fig. 2a shows the fluorescence obtained from a representative single wild-type baculovirus (BacWT) for GP64 (yellow) and the viral envelope (magenta) using a fluorophore that intercalates in the lipid bilayer (Methods). Fig. 2b illustrates the fluorescent signal obtained from a representative single BacE25-EGFP baculovirus for E25-EGFP (green) and the viral envelope (magenta). Finally, Fig. 2c shows an example of a single BacP24-EGFP baculovirus fluorescence obtained from P24-EGFP (green) and the envelope (magenta). Fig. 2d shows the mean±sd of the fluorescence intensity along single baculoviruses obtained from at least 15 independent virions. As illustrated in the graph, GP64 fluorescence (red line) is restricted to one pole of the baculovirus, while the fluorescence of the envelope (black line) covers a larger area as expected. We used the fluorescence from the envelope to delimit the baculovirus length, as the envelope is the outermost layer in viruses. Most interestingly, the fluorescence obtained from E25-EGFP (green line) follows that obtained with the envelope fluorescent marker (black line). This indicates that the envelope protein E25 distributes homogeneously along the envelope. Finally, the fluorescence obtained from P24-EGFP (blue line) resembles more of that obtained with GP64 (Fig. 2d).

### P24 nucleocapsid protein distributes in one pole of the baculovirus, near to GP64

**Fig. 3.**
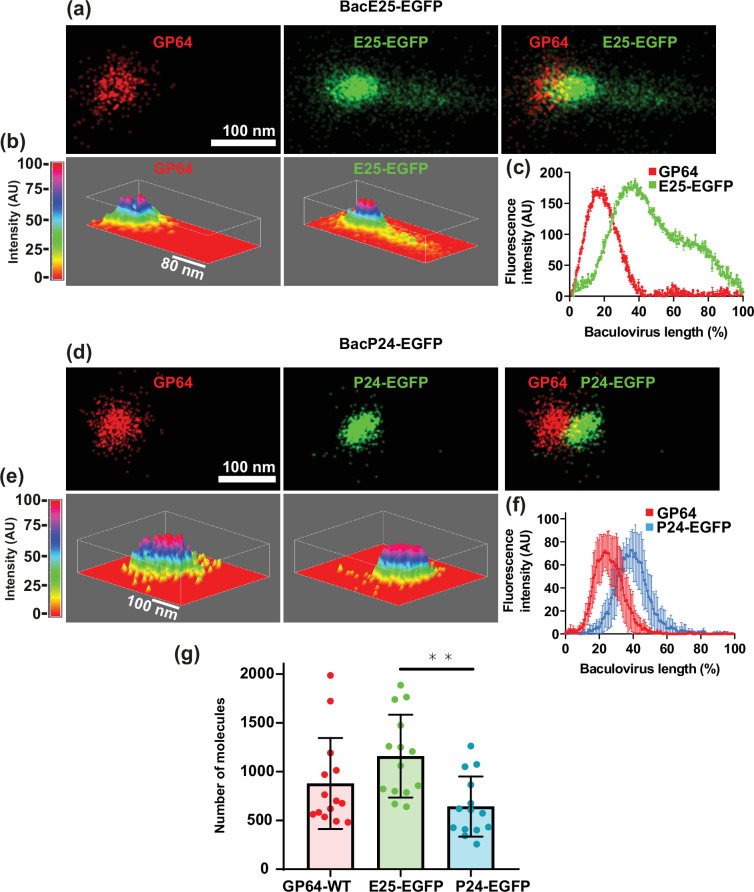
Spatial distribution of GP64, P24-EGFP and E25-EGFP proteins in BV in relation to GP64. Purified recombinant BV BacE25-EGFP (**a**) and BacP24-EGFP (**d**) were mounted on coverslips and immunolabelled to detect the GP64 protein. GP64 was used as a reference to determine the polarity and differential distribution of E25-EGFP and P24-EGFP. For single-molecule detection, we used the Nanoimager from ONI (Oxford Nanoimaging Ltd.). Excitation wavelengths were the same as in Fig. 2. 3D surface analysis of the fluorescence distribution of E25-EGFP (**b**) and P24-EGFP (**e**). The height of the plot maps the fluorescence intensity in arbitrary units. Average fluorescence distribution along the baculovirus length (mean±sd) for GP64/E25 EGFP (**c**) and GP64/P24 EGFP (**f**). Baculovirus lengths were normalized to percentages for easier comparison among individual viruses with different lengths. (g) Quantification of number of molecules present in individual baculoviruses for wild type (GP64-WT), recombinant E25-EGFP and P24-EGFP. Each dot represents the number of molecules in an individual baculovirus. Bars show the mean and standard deviation for each condition. **Indicate statistically significant different results.

To continue the study on the distribution of structural proteins on individual baculoviruses, we conducted experiments directed to identify the fluorescence distribution of GP64 and E25-EGFP on the same virion. Fig. 3a shows a representative example of the fluorescence obtained for both proteins. To illustrate more clearly the spatial distribution of both structural proteins, we plotted the fluorescence intensity in a 3D plot, where the height shows the intensity in arbitrary units (Fig. 3b). A line analysis of the fluorescence intensity over the baculovirus length shows clearly that the signal from GP64 is limited to one end of the baculovirus (Fig. 3c). As shown before (Fig. 2), the fluorescence from E25-EGFP projects further (Fig. 3c). These results further confirm the restricted localization of GP64, in agreement with what we and others have observed using TEM-immunogold (Fig. 1c). This result also shows that the expression of the recombinant protein E25-EGFP does not alter the localization of GP64, which is identical in wild-type baculovirus (Fig. 2) and recombinant BacE25-EGFP baculovirus (Fig. 3).

We proceeded next to analyse the localization of P24-EGFP with respect to GP64 (Fig. 3d). Unlike E25-EGFP, P24-EGFP shows a restricted localization like that obtained with GP64 (Fig. 3f), although the fluorescence intensity curve for P24-EGFP is slightly shifted to the right when compared to that obtained with GP64 (Fig. 3f).

SMLM studies not only provide precise distribution of proteins, one molecule at a time, but also allow the counting of the number of molecules present in the sample. We took advantage of this feature to determine the number of molecules present on individual baculoviruses for GP64, E25-EGFP and P24-EGFP (Fig. 3g). The molecule counting of at least 15 independent baculoviruses rendered a value of 878±466 (mean±sd) individual molecules of GP64 in wild-type baculovirus. Similar analysis showed values of 1158±424 molecules for E25-EGFP and 642±308 molecules for P24-EGFP (Fig. 3g).

### Assessment of the number of copies of P24 and recombinant P24-GFP on individual baculovirus

**Fig. 4.**
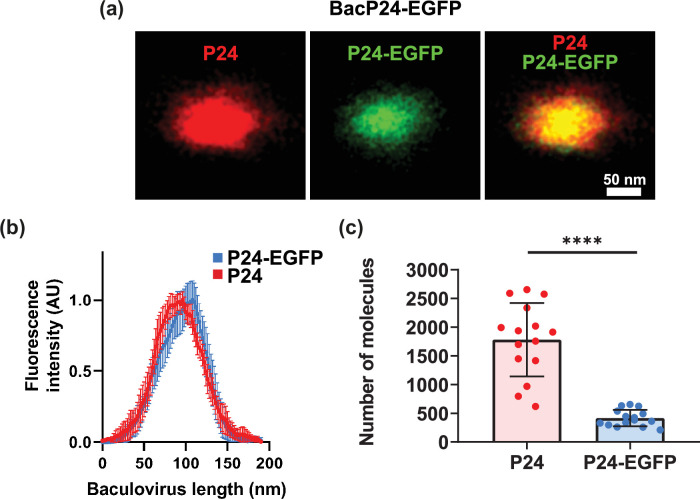
The polarized localization of P24 is not altered by the expression of recombinant structural proteins fused to EGFP. (**a**) Representative SMLM images of endogenous P24 labelling and BacP24-EGFP. (**b**) Average fluorescence distribution along the baculovirus length (mean±sd) for endogenous P24 and P24-EGFP on the same baculoviruses. Fluorescence intensity was normalized (from 0 to 1) and analysed using an unpaired t-test to compare the means of two independent groups and determine whether statistically significant differences existed. The result indicates that there is no difference between endogenous P24 and P24-EGFP distribution. (**c**) Single-molecule counting to determine the average number of endogenous P24 and recombinant P24-EGFP. Data shows mean±sd (*n*=14). Values are for endogenous P24=1781.26±639.45 and for P24-EGFP=416.26±144.19. All data were obtained from 15 different individual baculoviruses.

To continue the characterization of the spatial distribution of P24, we conducted experiments directed to determine if the distribution of endogenous P24 is like that of recombinant P24-EGFP (Fig. 4a). Using an antibody against endogenous P24, we could detect the same baculovirus P24 and P24-EGFP (Fig. 4a, left panel with signal in red). As illustrated in the representative image, endogenous P24 and recombinant P24-EGFP share the same location. This is evident also in the line plot compiling the fluorescence intensity from at least 15 independent baculoviruses (Fig. 4b).

Taking advantage of the capacity for single-molecule counting with SMLM studies, we analysed the number of total P24 and recombinant P24-EGFP molecules present in individual baculoviruses (Fig. 4c). For total P24 (endogenous+P24 EGFP), we obtained a value of 1781±639 molecules (mean±sd, *n*=15). For P24-EGFP, we obtained a value of 416±144 individual molecules (mean±sd, *n*=15). Since our antibody detects both the endogenous P24 and P24-EGFP, we can subtract the total P24 from the value obtained for P24-EGFP to obtain the endogenous number of P24 molecules. The result indicates that there are, on average, 1365 molecules of P24 per baculovirus.

### Gradients of structural proteins on individual baculovirus: a working model

**Fig. 5.**
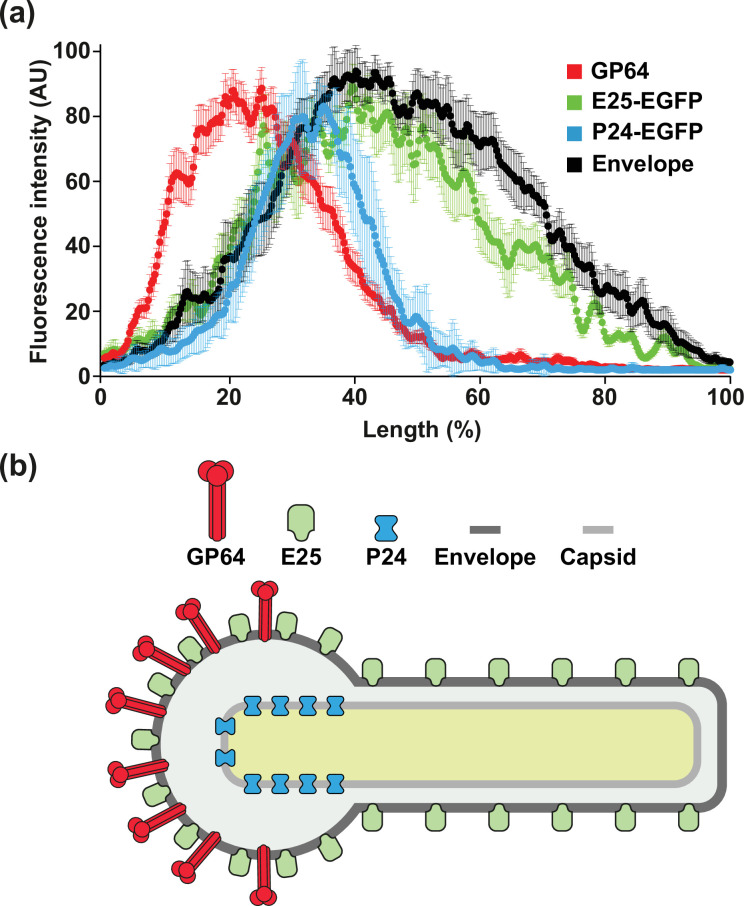
Differential distribution of GP64, E25 and P24 proteins in AcMNPV baculovirus. (**a**) Average fluorescence distribution along the baculovirus length of GP64, E25 and P24 proteins resolved by SMLM. Curves are from gathering all the data presented in the figures. The viral envelope was used to determine the spatial limits of each baculovirus. (**b**) Graphical representation of the differential localization of the structural envelope proteins GP64 and E25, as well as the nucleocapsid protein P24.

Combining the line profile plots to illustrate the fluorescence distribution along the baculovirus for the three proteins studied here provides a roadmap of their differential distribution (Fig. 5a). GP64 and P24 show a polarized distribution on one end of the baculovirus, while E25 appears to distribute throughout the viral envelope (Fig. 5a). Fig. 5b shows a cartoon of how the different structural proteins may arrange on the baculovirus.

## Discussion

Baculovirus is one of the most complex viruses found in nature [20]. Their life cycle presents two main forms, the occluded and BV [19]. Occluded virions are incorporated into a crystal structure formed mainly by a single protein known as polyhedrin [31]. Due to its geometry, this crystal structure is known as polyhedra, one of the most resilient protein structures known to date [31]. Polyhedra protects occluded baculovirus from damage from the environment, keeping virions infective for years at ambient temperature [24,31].

According to proteomics analysis, BVs are formed from at least 50 different structural proteins and a lipid bilayer known as the envelope [24]. From all these proteins identified in BV, only the precise localization of GP64 has been determined using TEM-immunogold [19,21,23]. The results presented here using SMLM confirm the TEM-immunogold findings. GP64 localization is restricted to one end of the baculovirus. The molecular mechanisms preventing GP64 from diffusing along the envelope remain obscure, but ongoing studies using SMLM may help to solve this conundrum.

GP64 is not the only protein polarized in baculovirus; here, we show for the first time that the nucleocapsid-associated protein P24 is also located at one end of the virus, sharing a similar location with GP64. Thus, polarized localization of proteins occurs at both the nucleocapsid (P24) and envelope (GP64). Worth noting, endogenous P24 and P24-EGFP show identical spatial distribution and polarization, and this indicates that the fusion of EGFP does not appear to alter P24 spatial distribution. We observed similar results regarding endogenous P24 distribution in baculovirus expressing E25-EGFP. P24 and GP64 show polarized distribution towards the same pole of the baculovirus. Future studies identifying the spatial distribution of other structural proteins may aid in determining if protein polarization occurs only at one end or both ends of the baculovirus.

Most interestingly, E25 (an envelope protein) shows a more homogenous distribution in the baculovirus, which is indistinguishable from the distribution obtained using a lipid fluorescent marker for the viral envelope. This result would be expected for an envelope protein that distributes homogeneously throughout the envelope.

Using SMLM, we have determined the number of single GP64, P24 and E25-GFP molecules present in a single baculovirus. GP64 has been previously identified as one of the most abundant proteins in BV; here, we show that each individual baculovirus carries, on average, 878 copies of this glycoprotein. Most surprisingly, our molecule counting analysis shows that each baculovirus displays about 1158 copies of E25-GFP; unfortunately, we could not assess the number of endogenous E25 since we do not have an antibody for this purpose, but we assume that the number of molecules will be greater, based on what we have found with P24. Using an in-house-produced antibody against P24, we were able to determine the total number of P24 molecules, which amounted to 1781, a number greater than that obtained for recombinant P24-GFP (416 copies in average). This is expected since the antibody recognizes both endogenous P24 and recombinant P24-GFP. Subtracting the total number of P24 molecules from P24-GFP results in 1365 molecules of endogenous P24. These results strongly suggest that both P24 and E25 are at least as abundant as GP64.

Even though TEM-immunogold provides information about the presence of a protein of interest in baculovirus, the number of gold particles distributed on the virion is limited and does not facilitate the precise assessment of the differential distribution of such protein on the surface of the virus. The use of SMLM in combination with molecular biology methods to generate recombinant baculovirus carrying fluorescent proteins fused to structural proteins provides a more accurate assessment of the differential distribution of a protein of interest. This is possible because we measure the fluorescence derived from all the individual molecules present on each virus (which may amount to hundreds, as mentioned above), unlike TEM-immunogold where we can detect only a handful of gold nanoparticles.

Another important question that remains unanswered is if the restricted localization of some structural proteins such as P24 may play a role in baculovirus infectivity or in protein functionality. Ongoing studies using SMLM in combination with mutagenesis of polarized structural proteins to force delocalization may provide insights into this matter.

## supplementary material

10.1099/jgv.0.002054 Uncited Supplementary Material 1.

## Abbreviations

AcMNPV: *Autographa californica nucleopolyhedrovirus*
BV: budded viruses
PALM: photo-activated localization microscopy
ROI: region of interest
SMLM: single-molecule localization microscopy
STORM: stochastic optical reconstruction microscopy
TEM: transmission electron microscopy
