# Supplementary material for: Resolving the differential distribution of structural proteins in baculovirus using single-molecule localization microscopy
Source: J Gen Virol. 2024 Dec 2;105(12):002054. doi: 10.1099/jgv.0.002054 (PMC11610606; doi:10.1099/jgv.0.002054)
Supplement: Uncited Supplementary Material 1. [file jgv-105-02054-s001.pdf]

Molecule counting

```
# Install the Readxl and ggplot libraries
# install.packages("readxl")

# Load the library
library(readxl)
library(ggplot2)
# Read the Excel file
datos_Bac <- read_excel("title.xlsx")

# Display the column names
column_names <- colnames(datos_Bac)
print(column_names)
# Convert the XCoordinates column to numeric type
datos_Bac$XCoordinates <- as.numeric(as.character(datos_Bac$XCoordinates))

# Convert the YCoordinates column to numeric type
datos_Bac$YCoordinates <- as.numeric(as.character(datos_Bac$YCoordinates))

# Round the XCoordinates and YCoordinates columns Y_Coordinates
data_bac$X_Coordinates <- round(data_bac$X_Coordinates, 6)
data_bac$Y_Coordinates <- round(data_bac$Y_Coordinates, 6)

# Filter values from multiple columns
columns_to_filter <- c("Channel", "X_Coordinates", "Y_Coordinates")
filtered_data <- data_bac[, columns_to_filter]

# Apply filter conditions
filtered_data <- data_bac[
filtered_data$Channel == # &
filtered_data$X_Coordinates > ### & filtered_data$X_Coordinates < ### &
filtered_data$Y_Coordinates > ### & filtered_data$Y_Coordinates < ###,
]
# Remove duplicate data based on columns XCoordinates and YCoordinates
filtered_data_without_duplicates <-
filtered_data[!duplicated(filtered_data[c("XCoordinates", "YCoordinates")]), ]

# If there are duplicates, take only one
filtered_data_without_duplicates <-
filtered_data_without_duplicates[!duplicated(filtered_data_without_duplicates[c("XCo
ordinates", "YCoordinates")]), ]
```

```
# Display the filtered data without duplicates
print(filtered_data_without_duplicates)
# Sum the number of rows
number_rows <- nrow(filtered_data_without_duplicates)
print(paste("Number of rows:", number_rows))

ggplot(filtered_data_without_duplicates, aes(x = XCoordinates, y = YCoordinates)) +
geom_point(size = 3, color = "green") +
labs(title = "Scatterplot", x = "XCoordinates", y = "YCoordinates") +
scale_y_reverse()
```
